# *Mycobacterium tuberculosis* Requires the ECF Sigma Factor SigE to Arrest Phagosome Maturation

**DOI:** 10.1371/journal.pone.0108893

**Published:** 2014-09-30

**Authors:** Stefano Casonato, Roberta Provvedi, Elisa Dainese, Giorgio Palù, Riccardo Manganelli

**Affiliations:** Department of Molecular Medicine, University of Padova, Padova, Italy; Bose Institute, India

## Abstract

SigE represents one of the best characterized alternative sigma factors of *Mycobacterium tuberculosis*, playing a major role in the response to several environmental stresses and essential for growth in macrophages and virulence. In previous work we demonstrated that a mutant of *M. tuberculosis* in which the *sigE* gene was disrupted by a cassette conferring hygromycin resistance is a promising vaccine candidate conferring better protection than *Mycobacterium bovis* BCG in a mouse model of infection. In this work we describe the construction of a new unmarked mutant in which the entire *sigE* gene was disrupted in order to fulfill the requirements of the Geneva consensus to enter clinical trials. After showing that the phenotype of this mutant is superimposable to that of the previous one, we further characterized the role of SigE in the *M tuberculosis* intracellular behavior showing that it is dispensable for replication in human pneumocytes, while it is essential for the arrest of phagosome maturation in THP-1-derived macrophages.

## Introduction

Tuberculosis (TB) represents a global public health problem and kills about 1.4 million people/year [1]. Current TB treatment requires a long and demanding multiple chemotherapy, while the only approved vaccine is the Bacille-Calmette-Guerin (BCG), an attenuated strain of *Mycobacterium bovis* that protects children from severe forms of TB, but not adults from pulmonary TB, failing to block the transmission chain [2]. The past decade has witnessed significant progress in the development of new TB vaccines, with the growth of a large and promising portfolio of candidates [3], but also the failure of the first phase II clinical trial of one of the most promising of them [4]. Moreover, the fundamental problem of the lack of reliable biomarkers to predict protection and disease progression still remained largely unsolved.

In a previous work we have characterized an *M. tuberculosis* H37Rv mutant in which the gene encoding the alternative sigma factor SigE was disrupted by the insertion of a hygromycin-resistance cassette [5]. SigE is subject to a very complex regulation at transcriptional and posttranslational level [6] and is involved in the transcription of several genes following exposure to different stress conditions damaging the bacterial surface [5]. As a consequence, the *sigE* knock out mutant was more sensitive to various surface-disrupting stresses, such as the detergent sodium dodecyl sulfate (SDS) and the antibiotic vancomycin, and to various oxidative compounds. Moreover, it was unable to grow in resting THP-1-derived macrophages and human dendritic cells, was more sensitive to killing from activated mouse macrophages, and was severely attenuated in mice [7], [8], [9], [10]. Due to its characteristics, we hypothesized that the *sigE* mutant could represent a promising live attenuated vaccine. Indeed, while its attenuation in mice was equal or better to that of BCG, it was more immunogenic and provided better protection from challenge with virulent *M. tuberculosis* [11].

In this work we describe the construction of a new H37Rv mutant in which the *sigE* gene was deleted without the introduction of a cassette conferring drug resistance, in order to fulfill the requirements to enter clinical trials [12]. After characterization of the new unmarked mutant, we provide proof that SigE is not required to growth in human pneumocytes, but is required to interfere with phagosome maturation.

## Materials and Methods

### Bacterial strains, growth media and transformation conditions

The following bacterial strains were used: *Escherichia coli* Top10 (Invitrogen), *E. coli* DH5α and *E. coli* HB101 (laboratory stocks), *M. tuberculosis* H37Rv (laboratory stock). *Escherichia coli* strains were grown at 37°C in Luria-Bertani (LB) broth or on LB agar plates. Mycobacterial strains were grown at 37°C in Middlebrook 7H9 broth (BD) in 150 ml roller bottles with slow rotation (3 rpm) or 7H10 agar plates (BD), supplemented with 0.2% glycerol and 0.05% Tween-80. For growth of *M. tuberculosis*, the medium was supplemented with 10% ADN (Albumin, Dextrose, NaCl). When needed, antibiotics were added to the media at the following concentrations: streptomycin (Sm): 20 µg/ml; kanamycin (Km): 50 µg/ml; hygromycin (Hyg) 150 µg/ml (*E. coli*) or 50 µg/ml (*M. tuberculosis*). Fluorescent mycobacteria for colocalization experiments were obtained by transformation with pMV10-25 [13].

### Construction of the *sigE* null mutant

**Figure 1:**
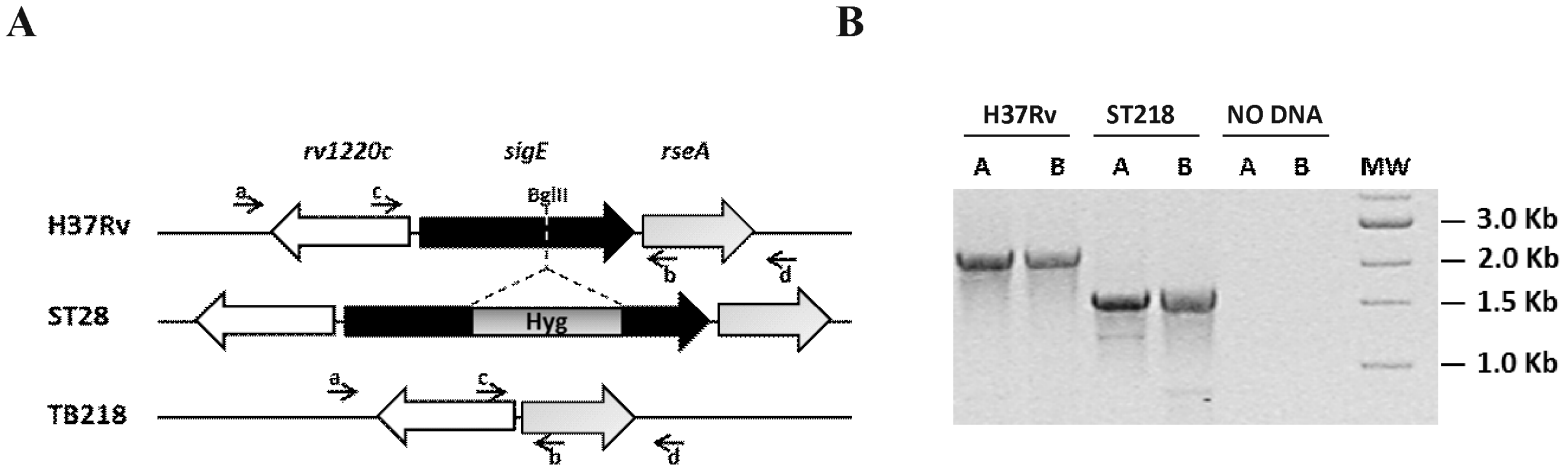
Construction of the *sigE* mutants. A) Schematic representation of *sigE*::hyg mutant (ST28) and of unmarked *sigE* mutant TB218. The Bgl II site used to disrupt the sigE gene in ST28 is indicated. B) Agarose gel of PCR products obtained amplifying H37Rv or TB218 genomic DNA with primers a–b (A) or c–d (B). Expected sizes of a–b PCR products were 2397 bp (H37Rv) and 1679 bp (ST218), while expected sizes of c–d PCR products were 2435 bp (H37Rv) and 1723 (ST218).

Deletion of the *sigE* gene (*rv1221*) has been obtained using the two-step homologous recombination method [14]. Primer pairs used to amplify the regions upstream and downstream of *rv1221* are shown in Table 1. Fragments, were sequentially cloned into p1NIL as ScaI/BglII and BglII/XhoI fragments respectively (restriction sites underlined in the primers) and the *lacZ*-*sacB*-*hyg* cassette from pGOAL19 was introduced as a *Pac*I fragment in the resulting vector to obtain the final suicide vector pSC42. This vector was electroporated into *M. tuberculosis* H37Rv and transformants were isolated on Km and Hyg. The occurrence of the single crossover was confirmed by PCR (data not shown). One mutant with the correct integration of pSC42 was grown in the absence of any drug to allow the second homologous recombination event to occur. Recombinants were isolated as white colonies on plates containing sucrose and X-gal. The occurrence of the double crossover leading to *rv1221* deletion was confirmed by PCR (Fig. 1) and by Southern blot (Fig. S1).

**Table 1:** Primer used in this study.

Primer	Sequence^a^	Purpose
RP1154	AGTACTGAATCCGTGAAGGCCGCGAACTT	*sigE* upstream region amplification
RP1155	AGATCTGCAAAGTTGCGATTCCGTATTCCCAA	*sigE* upstream region amplification
RP1556	AGATCTTCAACCCAGTTCGCTGAACTACTCAA	*sigE* downstream region amplification
RP1557	CTCGAGCTCCTGAAGCACCGGGTCAGCAA	*sigE* downstream region amplification
RP1204	TGCCATCAGCGGTGCGTAGG	Screening of *sigE* mutant (primer a Fig. 1B)
RP989	CTCGAGGTGGAACGGAACTGCC	Screening of *sigE* mutant (primer b Fig. 1B)
RP1207	GGCCGCCACCTTGGTGAACC	Screening of *sigE* mutant (primer c Fig. 1B)
RP990	AGGCCTGCATGTCGTCGTGTCC	Screening of *sigE* mutant (primer d Fig. 1B)

a Restriction sites are underlined in the primer sequence.

### Disk diffusion assay

*M. tuberculosis* strains were grown to early exponential phase. Aliquots of 100 µl containing 3×10^6^ cfu were spread on 7H10 plates. Paper disks (6.5 mm in diameter; Oxoid -Thermo Scientific) containing 10 µl of the inhibitory reagent were placed on top of the agar. Plumbagine, vancomycin and diamide were dissolved in H_2_O at a concentration of 10 mM, 10 mg/ml, and 10 mg/ml respectively. The diameters of the zones of inhibition were measured after 15 days of incubation at 37°C.

### Measurement of NO production by infected THP-1-derived macrophages cells

Supernatants were harvested from cultures of THP-1-derived macrophages infected at MOI of 10∶1 after 72 and 96 h. of infection. The production of NO was measured indirectly by assaying the presence of nitrite (NO_2_^−^) using the Griess reagent [15]. This was added 1∶1 to supernatants. Units of NO_2_^−^ were quantified from a standard curve using dilutions of NaNO_2_ (100 nM-100 µM) as a source of nitrite. Data were normalized to NO production in non-infected THP-1 cells. After 15 min at room temperature A_540_ values were read on a spectrophotometer. Mean values are the result of three independent experimental data ± SD, and asterisks indicate statistical significance (P<0.05) using Student's t-test.

### Cell cultures

The human monocytic cell line THP-1 was obtained from the ATCC collection (ATCC TIB 202). Monocytes were cultured at 37°C in a 5% CO_2_ atmosphere in RPMI-1640 medium (Life Technologies), supplemented with 2 mM L-glutamine and 10% fetal bovine serum. For subsequent experiments, THP-1 cell suspensions were adjusted to a concentration of 7.5×10^4^ cells/ml in warm supplemented RPMI, seeded in 96-well plates adding 100 µl of suspension per well, and cells were allowed to adhere and differentiate in the presence of 10 ng/ml phorbol 12-myristate 13-acetate (PMA) (Sigma), at 37°C in 5% CO_2_ atmosphere for 24 h.

The human lung adenocarcinoma epithelial cell line, A549 (gift of Prof. G. Delogu), was used as model of human type II alveolar epithelial cells. Cells were grown in complete medium consisting of RPMI-1640 supplemented with 10% fetal bovine serum (FBS), 2 mM L-glutamine and 5 µg/ml of gentamicin, and split when a confluent cell monolayer was obtained. A549 cells were seeded in 96-well plates and grown to 80–90% confluence.

### Infection of THP-1-derived macrophages and alveolar epithelial cell line A549

Prior the infection, the medium was removed; cells were extensively washed with serum-free RPMI and added with 100 µl of bacterial suspension from each strain (H37Rv, TB218, ST28 or complemented strain) in RPMI to obtain a multiplicity of infection (MOI) of 1∶20.

After 90 min of incubation at 37°C, the medium was removed and cells were washed twice with 100 µl of warm PBS to remove extracellular bacteria. The medium was replaced every 48 h. Every 24 h, the medium was removed from three wells, and then intracellular bacteria were released by lysing the cells with 100 µl of 0.05% SDS and immediately diluted in Middlebrook 7H9. In this condition viability of the mutant is not affected (data not shown). The number of viable intracellular bacteria was determined by plating serial dilutions of the lysis solution onto Middlebrook 7H10 agar supplemented with 10% OADC. About 95% of the cells remained viable during the entire experiment, as determined by trypan blue exclusion.

### Intracellular trafficking in THP-1-derived macrophages

Macrophages were seeded on glass coverslips and infected under the same conditions as the survival experiments Macrophages were infected as previously described at an MOI of 1∶1. After 48 h of infection, bacteria were fixed with 4% paraformaldehyde (PFA) in PBS at room temperature for 30 min. Finally, PFA was removed and cells were maintained in PBS at 4°C until their labeling. For LysoTracker labelling, cells were incubated at 37°C with 50 nM of LysoTracker (Molecular Probes) during 2 h, and subsequently washed, before fixing. Then, coverslips were permeabilized and incubated with a blocking solution of PBS, containing 2% FBS, 0.1% bovine serum albumin (BSA) and 1% saponin, for 30 min and incubated 1 h with primary antibodies against LAMP-1 or CD63. As primary antibodies we used monoclonal mouse anti-human LAMP-1 (Fitzgerald) diluted 1∶100 and monoclonal mouse anti-human CD63 (Fitzgerald) diluted 1∶100. After primary labeling, cells were washed three times with PBS-1% saponin, and incubated with fluorescent secondary antibodies in absence of light for 1 h. As secondary antibodies: we used Alexa Fluor 488 F(ab′)_2_ fragment of goat anti-rabbit IgG (Molecular Probes) and Alexa Fluor 594 F(ab′)_2_ fragment of goat anti-mouse IgG (Molecular Probes), both of them diluted 1∶100. Finally, cells were washed three times with PBS, coverslips were mounted in Fluoromount-G (Southern Biotech) on a microscope slide, and let at 4°C overnight. Indirect immunofluorescence was examined on a Leica TCSNT/SP2 confocal microscope (Leica Microsystem) through an ×63 oil immersion objective (Plan Neofluar). To calculate the percentage of colocalization for each coverslip, the superposition of fluorescence for a minimum of 100 internalized isolated bacteria was analyzed. At least two slides were analyzed in blind from each of three independent infections.

## Results

### Construction of an unmarked *sigE* null mutant

In a previous work we described the construction of an *M. tuberculosis sigE* mutant strain (ST28) obtained inserting a cassette conferring hygromycin resistance into a unique BglII site internal to the *sigE* structural gene *rv1221* [5]. This strain has proven to be a good vaccine candidate being severely attenuated in mice and inducing a strong immunological response leading to better protection than BCG in a mouse model of infection [11]. In order to fulfill one of the Geneva consensus requirements to enter human clinical trials [12], we created a new mutant in which the entire gene was deleted without the insertion of any antibiotic-resistance cassette. In order to reach this goal we followed a well-established two-step homologous recombination method [14] to delete the entire *rv1221* from the chromosome of *M. tuberculosis* H37Rv (see Materials and Methods section). Two mutants were obtained in which deletion of *rv1221* was confirmed by PCR (Fig. 1) and Southern blot (Fig. S1). One of them, named TB218, was selected for further studies.

Genetic complementation of the new mutant was obtained by electroporation of an integrative plasmid containing a wild type copy of *rv1221* and its promoter region [5], generating the strain TB382. The reintroduction of a copy of *sigE* in the chromosome of the complemented strain was confirmed by PCR (data not shown).

### Characterization of the unmarked *sigE* null mutant TB218

No significant differences were observed between the growth in axenic culture of the new unmarked mutant (TB218) and the old one (ST28), while both strains showed a slightly longer lag phase respect to the wt strain (data not shown). ST28 was previously shown to be highly sensitive to surface and oxidative stress, to be unable to replicate in THP-1-derived macrophages and to have a strongly reduced level of basal expression of *sigB* a gene encoding a primary, non-essential sigma factor [5]. In order to evaluate if the new unmarked *sigE* mutant had the same characteristics, we measured the sensitivity of H37Rv, TB218 and the complemented strain TB382 to the antibiotic vancomycin, the superoxide generator plumbagine and the thiol-specific oxidizing agent diamide using a disk diffusion assay. As shown in Table 2, TB218 was more sensitive than H37Rv to the three compounds, and the wild type phenotypes were fully restored in the complemented strain TB382. Sensitivity to 0.05% SDS was assessed in liquid culture and also in this case TB218 was shown to be more sensitive than parental and complemented strain to this surface-stressing compound (data not shown). Moreover, we also showed by quantitative RT-PCR that, as in TB28, *sigB* expression level in TB218 was about 10 folds lower than that found in the parental and complemented strains (data not shown).

**Table 2:** Sensitivity of H37Rv, TB218 and TB328 to various stressing compounds^a^.

	Strain		
	H37Rv	TB218	TB382
**Plumbagine**	3.6±0.1^b^	4.6±0.1	3.9±0.1
**Vancomycin**	5.1±0.1	6.25±0.25	5.0±0.5
**Diamide**	0.3±0.1	3.5±0.4	0.3±0.1

a Disk diffusion assay.

b The reported values represent the average the standard deviation of the diameter of the inhibition zone in cm. The experiment, performed in triplicate, was repeated twice with independent bacterial cultures.

**Figure 2:**
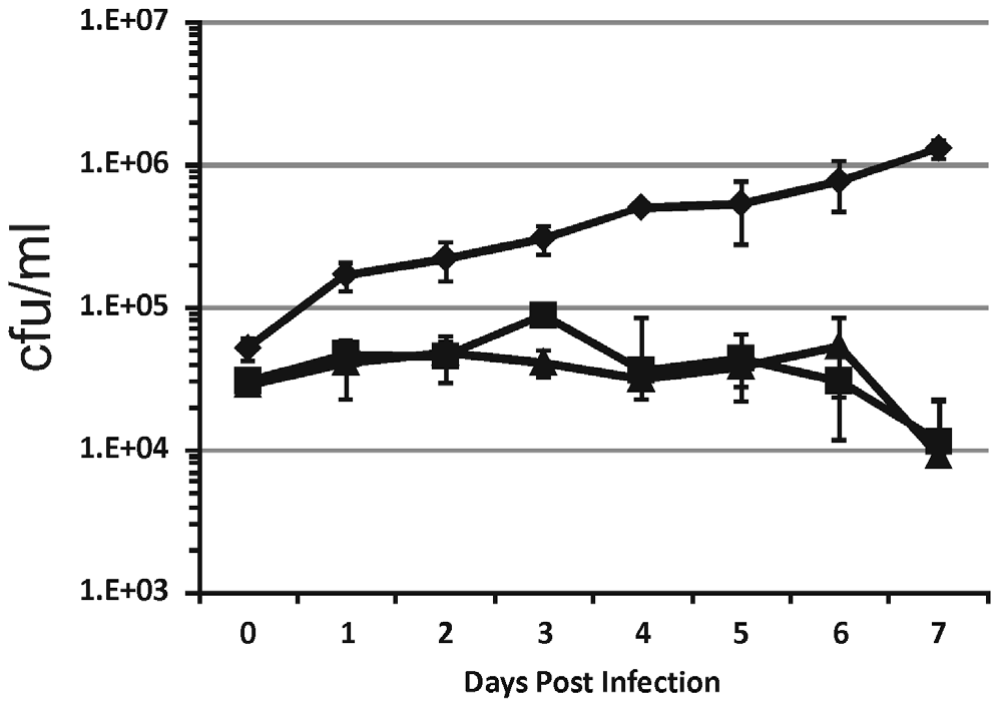
Growth of *M. tuberculosis* H37Rv and of the *sigE* TB218 mutant in THP-1-derived macrophages. The results are expressed as cfu/ml. The reported values represent the average and the standard deviation obtained from three parallel independent infections. The experiment was repeated twice with different bacterial and THP-1 cultures. H37Rv (diamonds), TB218 (triangles), TB328 (squares).

Finally, we evaluated the growth of TB218 in human macrophages; at this purpose THP-1-derived macrophages were infected at an MOI 1∶20, with H37Rv, ST28, or TB218. As shown in Figure 2, the intracellular growth of the two *sigE* mutants was comparable, but strongly impaired in respect to that of H37Rv.

Taken together, these data clearly showed that, as expected, the new unmarked *sigE* null mutant TB218 displays the same phenotype of the previous *sigE* mutant ST28.

### NO production from THP-1-derrived infected macrophages

**Figure 3:**
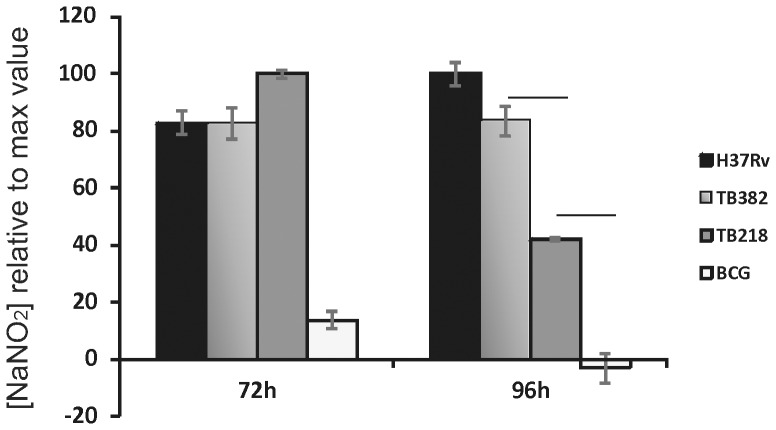
Nitric oxide production by *M. tuberculosis*-infected THP-1-derived macrophages. Cells were infected at an MOI of 10∶1. The production of NO was measured indirectly by assaying the presence of nitrite (NO_2_^−^) using the Griess reagent. Mean values are the result of three independent experimental data ± SD, and asterisks indicate statistical significance (P<0.05) using Student's t-test.

**N**itric oxide (NO) production represents one of the mechanisms used by macrophages to control *M. tuberculosis* [16] and to induce bacterial dormancy [17], even if contrasting evidences has been reported [18]. Since in previous experiments the *sigE* mutant was shown to stimulate a different kind of inflammatory response both in vitro and in vivo [9], [11], [19], we decided to evaluate its ability to stimulate NO production after infection of THP-1-derived macrophages. At this purpose the level of NO production following infection with TB218 or its wild type parental strain, was measured in the supernatants of THP-1-derived macrophages after infection with H37Rv, TB218, its complemented strain or BCG. Figure 3 clearly shows that after 72 h from the infection, the *sigE* mutant TB218 was able to induce an NO production 4 times higher than that induced by BCG and comparable with that induced from its parental and complemented strains. After further 24 h of incubation, while the amount of NO in H37Rv and complemented strain-infected cells remained constant, that in *sigE* mutant-infected cells was significantly reduced, even if still significantly higher than that present in BCG-infected cells.

### SigE is not required for *M. tuberculosis* replication in alveolar epithelial cells

**Figure 4:**
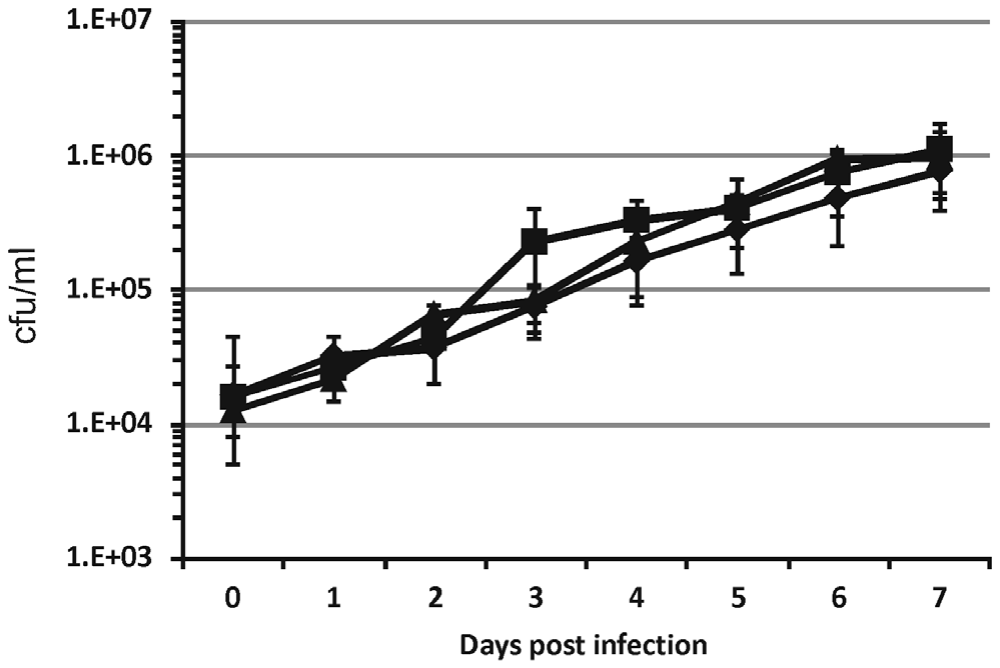
Growth of *M. tuberculosis* H37Rv and of the *sigE* TB218 mutant in alveolar epithelial cells A549. The results are expressed as cfu/ml. The reported values represent the average and the standard deviation obtained from three parallel independent infections. The experiment was repeated twice with different bacterial and A549 cultures. H37Rv (diamonds), TB218 (triangles), TB328 (squares).

Although *M. tuberculosis* primarily infects alveolar macrophages after reaching the lungs, it can also infect alveolar epithelial cells (pneumocytes) [20]. Since no information regarding the role of SigE in pneumocytes infection is available, we examined the ability of TB218 and ST28 to invade and replicate in the human type II alveolar epithelial cells A549. As shown in Figure 4, no difference in the intracellular replication rate among the three strains was observable over the eight days of infection demonstrating that SigE is not required for *M. tuberculosis* growth in human pneumocytes.

### Altered trafficking of the *sigE* mutant in THP-1-derived macrophages

**Figure 5:**
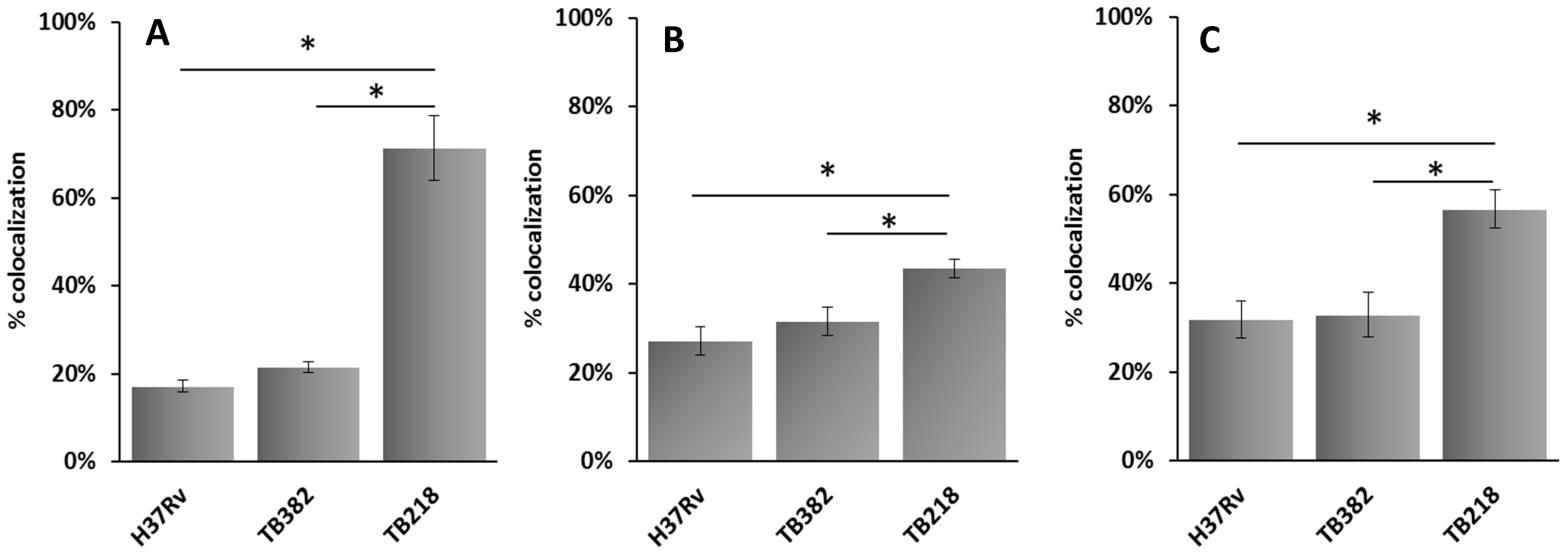
Confocal microscopy analysis. THP-1-derived macrophages were infected with GFP-expressing H37Rv, TB218 or TB238 at an MOI of 1∶1. After 48 h of infection, cells were treated with LysoTracker or through immunofluorescence using anti CD63 or anti LAMP-1 antibody. Data were express as the percentage of mycobacteria inside labelled compartments respect to internalized mycobacteria. Mean values are the result of three independent experimental data ± SD, and asterisks indicate statistical significance (P<0.05) using Student's t-test. A) LysoTracker: B) CD63; C) LAMP-1.

Due to the differences observed between the *sigE* mutant TB218 and its wild-type parental strain in their ability to grow inside macrophages, we sought to examine whether a significant alteration in the intracellular trafficking was evident in the mutant strain. At this purpose we analyzed by immunofluorescence the colocalization of GFP-expressing variants of H37Rv, TB218, and TB382, with acidified compartments stained with LysoTracker. After 48 hours of infection 71% of the *sigE* mutants colocalized with LysoTracker, while the colocalization of GFP-expressing H37Rv and TB382 was 17 and 21.5%, respectively (Fig. 5A and S2). For a better characterization, we also studied colocalization with two membrane glycoproteins typical of late-compartments of the phagocytic route (LAMP-1 and CD63). As shown in Figures 5B–C and Figures S3 and S4, the *sigE* mutant colocalized with LAMP-1 and CD63 more than H37Rv and the complemented strain [56.7% *vs* 31.8% and 32.9% (LAMP-1) and 43.5% *vs* 27% and 31.5% (CD63), respectively] confirming the involvement of SigE in the regulation of the pathway used by *M. tuberculosis* to manipulate phagolysosome maturation.

## Discussion

Despite the global incidence of TB has been slowly diminished over the last decade *M. tuberculosis* is second only to HIV in deaths caused by a single pathogen worldwide [1]. TB continues to persist regardless of the widespread use of BCG, the only licensed vaccine to prevent TB. BCG's limited efficacy coupled with the emergence of drug-resistant strains of *M. tuberculosis* emphasizes the need for a more effective vaccine for combating this disease [2].

Among the promising candidates for the development of a new vaccine against TB, we recently described an attenuated strain of *M. tuberculosis* in which the gene encoding the ECF sigma factor SigE was disrupted [21]. This mutant is highly attenuated permitting complete survival of the infected animals after 4 months of infection, with significantly lower bacillary loads and less tissue damage compared to animals infected with the parental or complemented strains [11].

Moreover, the *sigE* mutant is highly attenuated in SCID mice [11], produces a significantly lower mortality than BCG in nude mice and induces better protection, allowing 80% mouse survival after 4 months of challenge with an hypervirulent *M. tuberculosis* Beijing isolate [11].

Taken together, these observations suggest that the *sigE* mutant is safer and more immunogenic than BCG and justify the hypothesis that the *sigE* mutant could have strong potential as a novel attenuated vaccine.

In April 2009, the second Geneva Consensus has been held at WHO headquarters, to discuss issues regarding the regulatory requirements for live TB vaccines to enter Phase I trials, in particular those based on attenuated *M. tuberculosis* [12]. Particular attention was paid to the characterization and safety package likely to be required, including the issues of attenuation, the presence of antibiotic resistance markers in live vaccines and the nature of any attenuated vaccine phenotype. As a first step to accomplish the Geneva consensus requirements to enter human clinical trials we created a new version of the attenuated *sigE* mutant producing an unmarked deletion of the entire *sigE* gene (TB218). Moreover, we presented evidences that the new mutant is phenotypically equivalent to the previous mutant ST28, in which the *sigE* gene was disrupted by a cassette conferring Hyg resistance.

TB218 was also used to further characterize the role of SigE in *M. tuberculosis* virulence analyzing its role in intracellular growth in human pneumocytes, and in induction of NO production after macrophage infection. Our results clearly showed that TB218 during the first 72 h of infection was able to induce macrophages to produce NO at the same level of wild type *M. tuberculosis*. However, after further 24 h of infection its ability to induce NO production decreased significantly respect to that of the wild type strain, even if remaining higher than that of BCG (Fig. 3). The reason of this decrease cannot be simply explained with the inability of the *sigE* mutant to replicate intracellularly and need further investigation, since an *M. tuberculosis* mutant lacking *phoP*, even if unable to replicate inside macrophages, was shown to induce NO level comparable with that induced by its wild type parental strain up to five days post-infection [15], [22]. Finally, despite its inability to grow in human macrophages, the *sigE* mutant was shown to be able to replicate in human pneumocytes at the same rate of its wild type parental strain, suggesting that the SigE role is restricted to the interaction with professional phagocytic cells in response to the strong stress encountered in these cells.

SigE has been previously shown to regulate genes whose products are involved in mycolic acid biosynthesis and fatty acid degradation, as well as in membrane protein quality control and membrane stabilization [19]. Since surface proteins and lipids are good candidates for effectors involved in the arrest of phagosome maturation typical of *M. tuberculosis*, we used immunocytochemistry to examine the intracellular trafficking of TB218 in THP-1-derived macrophages. First of all we analyzed the colocalization of TB218 with acidic compartments taking advantage of the acidotropic characteristics of LysoTracker and showed that while as expected H37Rv largely avoided phagosome acidification [23], [24], the *sigE* mutant localized in a significantly higher proportion of acidified compartments, and this phenotype was reversed in the complemented strain (Fig. 5A). Thus, in contrast to its parental strain, TB218 is defective in blocking the phagosome acidification.

We could also show that TB218 colocalized slightly, but significantly more with the markers of phagolysosome fusion CD63 and LAMP-1 (Fig. 5B–C) strongly suggesting that TB218-containing phagosomes are more phagolysosome-fusion competent compared to those containing the wild type or the complemented strain. Since phagolysosome fusion leads to degradation of mycobacterial antigens, facilitating their presentation through the MHC-II pathway [24], [25], [26], the higher phagolysosome-fusion competence of TB218 phagosomes might at least in part explain the higher immunogenicity of the previous *sigE* mutant [11]. Further studies are needed to validate this hypothesis. Interestingly, TB218 shares these features with a *phoP* mutant of *M. tuberculosis* which represents the first attenuated *M. tuberculosis* strain entering phase I clinical trial [15], [22].

Concluding, our study shows that deletion of *sigE*, beyond rendering the bacterium sensitive to several environmental stresses, leads to an altered intracellular behavior in macrophages, with a decreased ability to arrest phagosome acidification and (to a lower extent) fusion with lysosomes. This altered intracellular trafficking might result in a more efficient processing from macrophages, and ultimately determine the efficacy of this mutant strain as a vaccine.

## Supporting Information

Figure S1 **Confirmation of** ***sigE*** **deletion by Southern-blot analyses.** (A) Schematic representation of the *sigE* DNA region in H37Rv. Gray boxes indicate the fragments upstream and downstream *sigE* amplified by PCR present in pSC42, the suicida plasmid used to construct the *sigE* null mutant TB218, SphI restriction sites are indicated. (B) 2.5 µg of H37Rv and TB218 chromosomal DNA were cut with SphI and run on a 0.8% TAE 1× agarose gel. The DNA was then ransfered on a nylon membrane and hybridized with a digoxigenin labeled probe containing both *sigE* upstream and downstream regions (shown in gray in panel A). DNA from H37Rv showed the expected bands whose predicted size was 855 and 2716 (red and yellow arrows, respectively). DNA from TB218 also showed two reactive bands: as expected the lower one was compatible with a size of 855 bp (red arrow), while the other was compatible with the expected size resulting from the deletion of the 706 bp of the *sigE* gene (2010 bp, green arrow). M: DNA molecular weight marker III digoxigenin labeled (Roche)
(PPTX)

Figure S2 **Confocal microscopy analysis of LysoTraker-stained infected THP-1-derived macrophages.** Representative images of cells infected with GFP expressing H37Rv, TB218 (*sigE* mutant) or TB382 (complemented strain) at an MOI of 1∶1. After 48 h of infection, cells were stained with LysoTraker (red). Colocalization of both red and green fluorescence indicates that the mycobacteria reside in acidic compartments. The overlap is demonstrated in the merged images, where yellow indicates a positive correlation. Boxed areas show enlargements of sections of interest with examples of negative (H37Rv and TB318) and positive (TB218) colocalization. Images were taken with a Leica T CSNT/SP2 confocal microscope using a ×63 oil immersion objective. To calculate the percentage of colocalization for each coverslip, the superposition of fluorescence for a minimum of 100 internalized isolated bacteria was analyzed. At least two slides were analyzed from each of three independent infections.
(PPTX)

Figure S3 **Confocal microscopy analysis of mycobacterial intracellular localization in human macrophages.** Representative images of CD63-stained THP-1-derived macrophages infected with GFP expressing H37Rv, TB218 or and TB382 at an MOI of 1∶1. After 48 h of infection, CD63 compartments were stained in red fluorescence using anti-CD63 antibodies. Colocalization of both red and green fluorescence indicates that the mycobacteria reside in CD63-associated compartments. The overlap is demonstrated in the merged images, where yellow indicates a positive correlation. Boxed areas show enlargements of sections of interest with examples of negative (H37Rv and TB318) and positive (TB218) colocalization. Images were taken with a Leica T CSNT/SP2 confocal microscope using a ×63 oil immersion objective. To calculate the percentage of colocalization for each coverslip, the superposition of fluorescence for a minimum of 100 internalized isolated bacteria was analyzed. At least two slides were analyzed from each of three independent infections.
(PPTX)

Figure S4 **Confocal microscopy analysis of mycobacterial intracellular localization in human macrophages.** Representative images of LAMP-1-stained THP-1-derived macrophages infected with GFP expressing H37Rv, TB218 or TB382 at an MOI of 1∶1. After 48 h of infection, LAMP-1 compartments were stained in red using anti LAMP-1. Colocalization of both red and green fluorescence indicates that the mycobacteria reside in LAMP-1-associated compartments. The overlap is demonstrated in the merged images, where yellow indicates a positive correlation. Boxed areas show enlargements of sections of interest with examples of negative (H37Rv and TB318) and positive (TB218) colocalization. Images were taken with a Leica T CSNT/SP2 confocal microscope using a ×63 oil immersion objective. To calculate the percentage of colocalization for each coverslip, the superposition of fluorescence for a minimum of 100 internalized isolated bacteria was analyzed. At least two slides were analyzed from each of three independent infections.
(PPTX)
