# Correction: *Mycobacterium tuberculosis* Requires the ECF Sigma Factor SigE to Arrest Phagosome Maturation

**DOI:** 10.1371/journal.pone.0115990

**Published:** 2014-12-15

**Authors:** The PLOS ONE Staff

Figure 3 is incorrect. The asterisks indicating statistical significance were inadvertently left out. The authors have provided a corrected version here.

**Figure 3:**
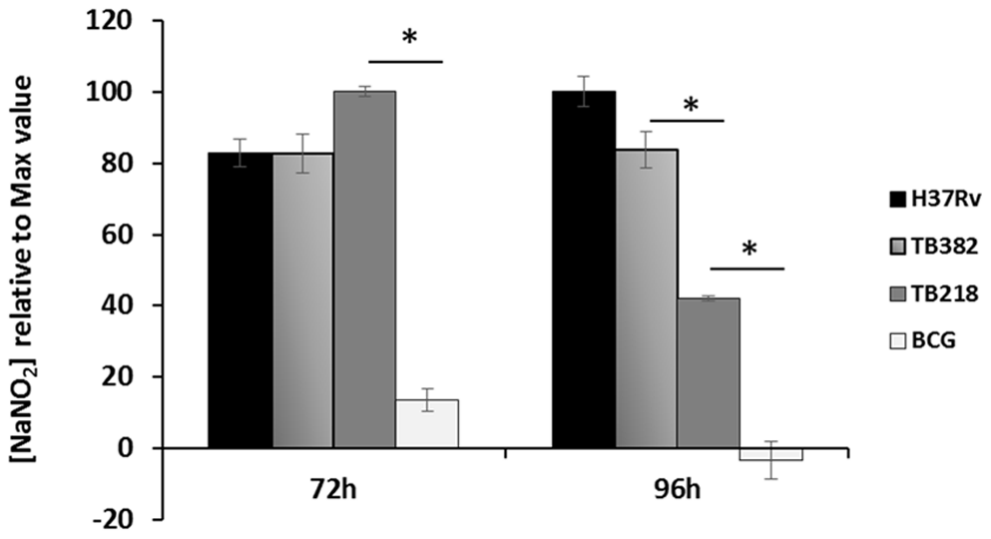
Nitric oxide production by *M. tuberculosis*-infected THP-1-derived macrophages. Cells were infected at an MOI of 10:1. The production of NO was measured indirectly by assaying the presence of nitrite (NO_2_−) using the Griess reagent. Mean values are the result of three independent experimental data ± SD, and asterisks indicate statistical significance (P<0.05) using Student's t-test.
