# Supplementary material for: Mycobacterium tuberculosis Requires the ECF Sigma Factor SigE to Arrest Phagosome Maturation
Source: PLoS One. 2014 Sep 30;9(9):e108893. doi: 10.1371/journal.pone.0108893 (PMC4182583; doi:10.1371/journal.pone.0108893)
Supplement: Figure S1 — Confirmation of sigE deletion by Southern-blot analyses. (A) Schematic representation of the sigE DNA region in H37Rv. Gray boxes indicate the fragments upstream and downstream sigE amplified by PCR present in pSC42, the suicida plasmid used to construct the sigE null mutant TB218, SphI restriction sites are indicated. (B) 2.5 µg of H37Rv and TB218 chromosomal DNA were cut with SphI and run on a 0.8% TAE 1× agarose gel. The DNA was then ransfered on a nylon membrane and hybridized with a digoxigenin labeled probe containing both sigE upstream and downstream regions (shown in gray in panel A). DNA from H37Rv showed the expected bands whose predicted size was 855 and 2716 (red and yellow arrows, respectively). DNA from TB218 also showed two reactive bands: as expected the lower one was compatible with a size of 855 bp (red arrow), while the other was compatible with the expected size resulting from the deletion of the 706 bp of the sigE gene (2010 bp, green arrow). M: DNA molecular weight marker III digoxigenin labeled (Roche) (PPTX) [file pone.0108893.s001.pptx]

## Slide 1
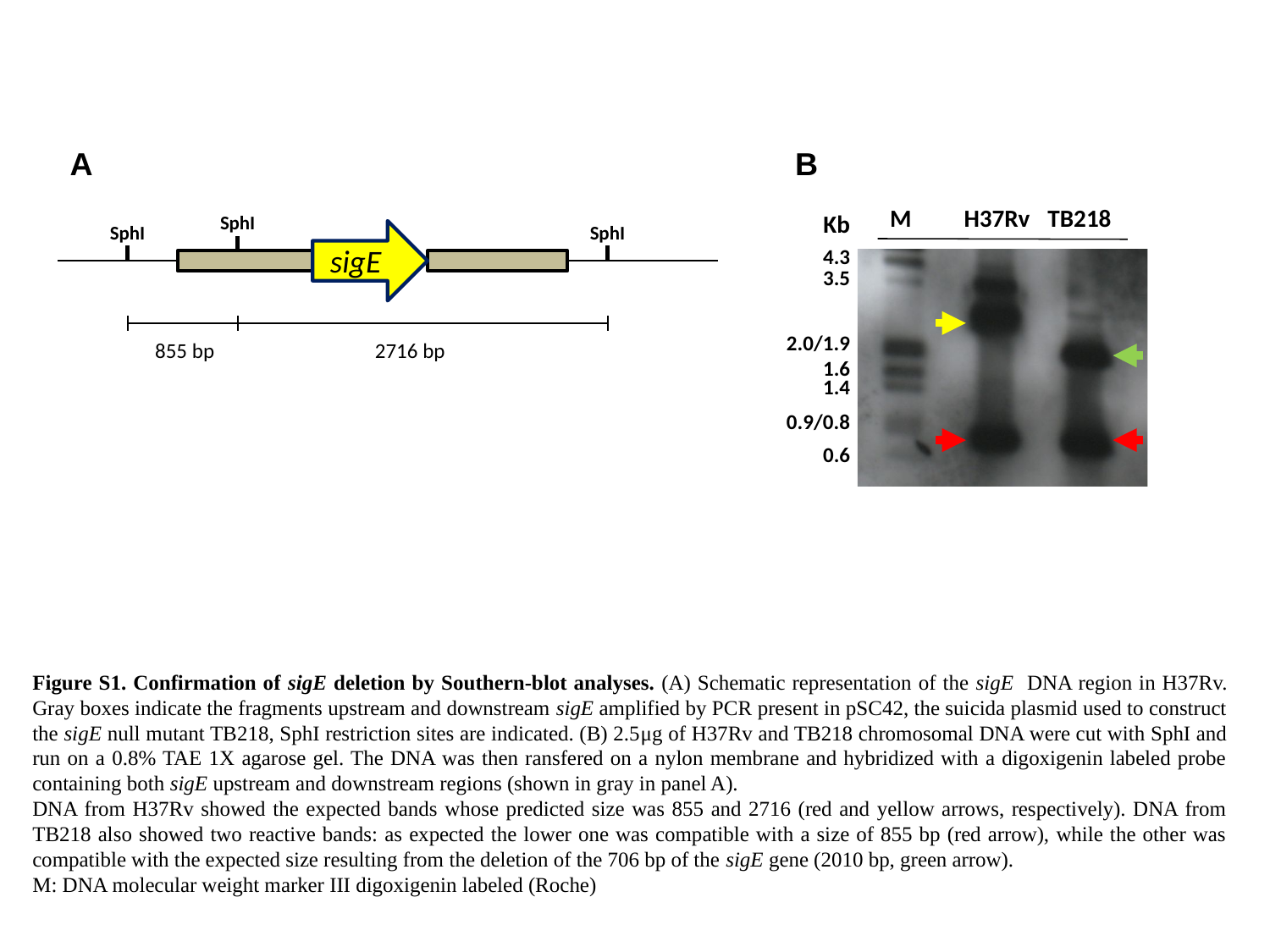

A
SphI
SphI
SphI
sigE
855 bp
2716 bp
B
M
H37Rv
TB218
Kb
4.3
3.5
2.0/1.9
1.6
1.4
0.9/0.8
0.6
Figure S1. Confirmation of sigE deletion by Southern-blot analyses. (A) Schematic representation of the sigE DNA region in H37Rv. Gray boxes indicate the fragments upstream and downstream sigE amplified by PCR present in pSC42, the suicida plasmid used to construct the sigE null mutant TB218, SphI restriction sites are indicated. (B) 2.5μg of H37Rv and TB218 chromosomal DNA were cut with SphI and run on a 0.8% TAE 1X agarose gel. The DNA was then ransfered on a nylon membrane and hybridized with a digoxigenin labeled probe containing both sigE upstream and downstream regions (shown in gray in panel A).
DNA from H37Rv showed the expected bands whose predicted size was 855 and 2716 (red and yellow arrows, respectively). DNA from TB218 also showed two reactive bands: as expected the lower one was compatible with a size of 855 bp (red arrow), while the other was compatible with the expected size resulting from the deletion of the 706 bp of the sigE gene (2010 bp, green arrow).
M: DNA molecular weight marker III digoxigenin labeled (Roche)
