# Supplementary material for: Mycobacterium tuberculosis Requires the ECF Sigma Factor SigE to Arrest Phagosome Maturation
Source: PLoS One. 2014 Sep 30;9(9):e108893. doi: 10.1371/journal.pone.0108893 (PMC4182583; doi:10.1371/journal.pone.0108893)
Supplement: Figure S2 — Confocal microscopy analysis of LysoTraker-stained infected THP-1-derived macrophages. Representative images of cells infected with GFP expressing H37Rv, TB218 (sigE mutant) or TB382 (complemented strain) at an MOI of 1∶1. After 48 h of infection, cells were stained with LysoTraker (red). Colocalization of both red and green fluorescence indicates that the mycobacteria reside in acidic compartments. The overlap is demonstrated in the merged images, where yellow indicates a positive correlation. Boxed areas show enlargements of sections of interest with examples of negative (H37Rv and TB318) and positive (TB218) colocalization. Images were taken with a Leica T CSNT/SP2 confocal microscope using a ×63 oil immersion objective. To calculate the percentage of colocalization for each coverslip, the superposition of fluorescence for a minimum of 100 internalized isolated bacteria was analyzed. At least two slides were analyzed from each of three independent infections. (PPTX) [file pone.0108893.s002.pptx]

## Slide 1
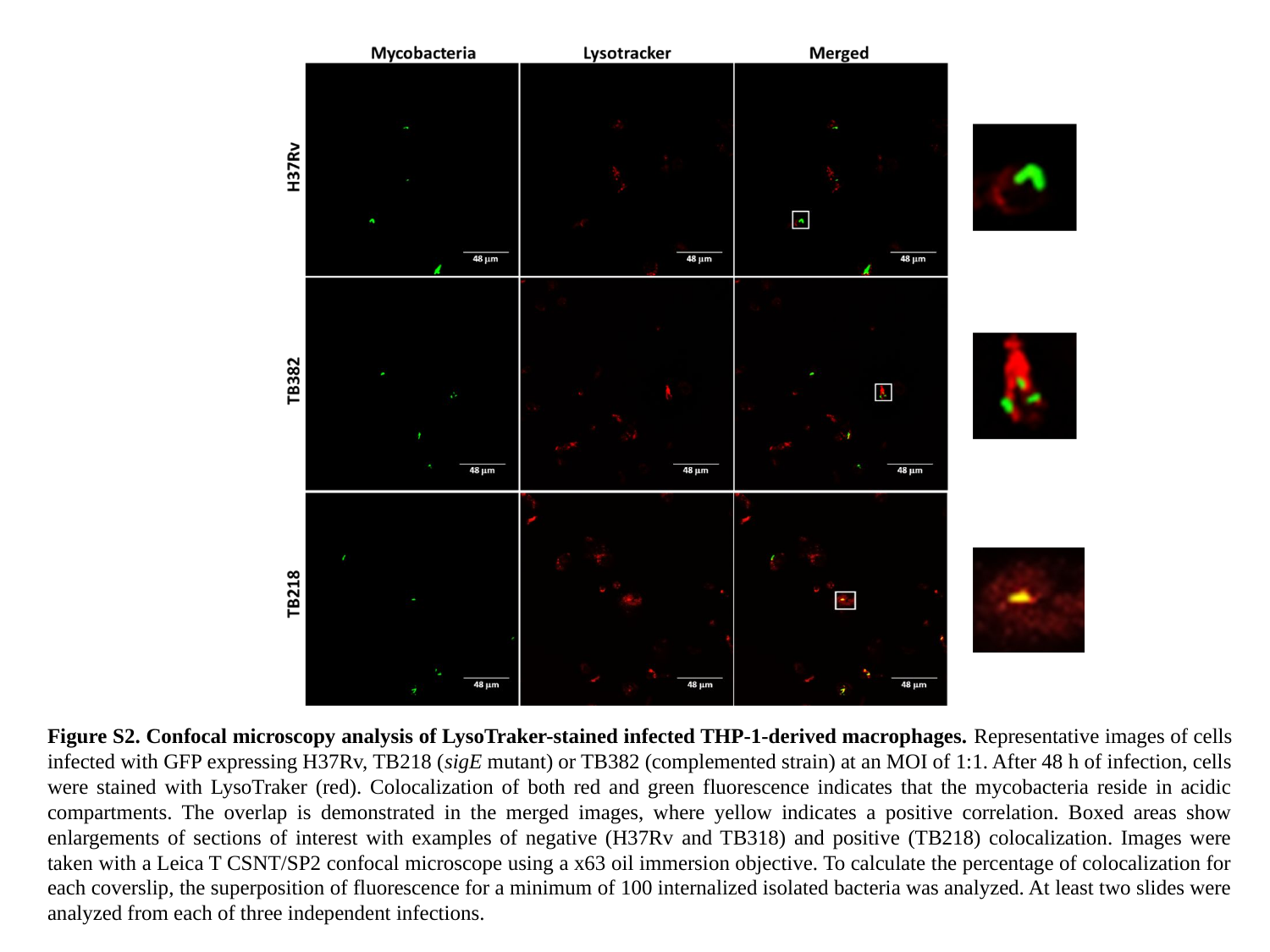

Figure S2. Confocal microscopy analysis of LysoTraker-stained infected THP-1-derived macrophages. Representative images of cells infected with GFP expressing H37Rv, TB218 (sigE mutant) or TB382 (complemented strain) at an MOI of 1:1. After 48 h of infection, cells were stained with LysoTraker (red). Colocalization of both red and green fluorescence indicates that the mycobacteria reside in acidic compartments. The overlap is demonstrated in the merged images, where yellow indicates a positive correlation. Boxed areas show enlargements of sections of interest with examples of negative (H37Rv and TB318) and positive (TB218) colocalization. Images were taken with a Leica T CSNT/SP2 confocal microscope using a x63 oil immersion objective. To calculate the percentage of colocalization for each coverslip, the superposition of fluorescence for a minimum of 100 internalized isolated bacteria was analyzed. At least two slides were analyzed from each of three independent infections.
