# Supplementary material for: Mycobacterium tuberculosis Requires the ECF Sigma Factor SigE to Arrest Phagosome Maturation
Source: PLoS One. 2014 Sep 30;9(9):e108893. doi: 10.1371/journal.pone.0108893 (PMC4182583; doi:10.1371/journal.pone.0108893)
Supplement: Figure S4 — Confocal microscopy analysis of mycobacterial intracellular localization in human macrophages. Representative images of LAMP-1-stained THP-1-derived macrophages infected with GFP expressing H37Rv, TB218 or TB382 at an MOI of 1∶1. After 48 h of infection, LAMP-1 compartments were stained in red using anti LAMP-1. Colocalization of both red and green fluorescence indicates that the mycobacteria reside in LAMP-1-associated compartments. The overlap is demonstrated in the merged images, where yellow indicates a positive correlation. Boxed areas show enlargements of sections of interest with examples of negative (H37Rv and TB318) and positive (TB218) colocalization. Images were taken with a Leica T CSNT/SP2 confocal microscope using a ×63 oil immersion objective. To calculate the percentage of colocalization for each coverslip, the superposition of fluorescence for a minimum of 100 internalized isolated bacteria was analyzed. At least two slides were analyzed from each of three independent infections. (PPTX) [file pone.0108893.s004.pptx]

## Slide 1
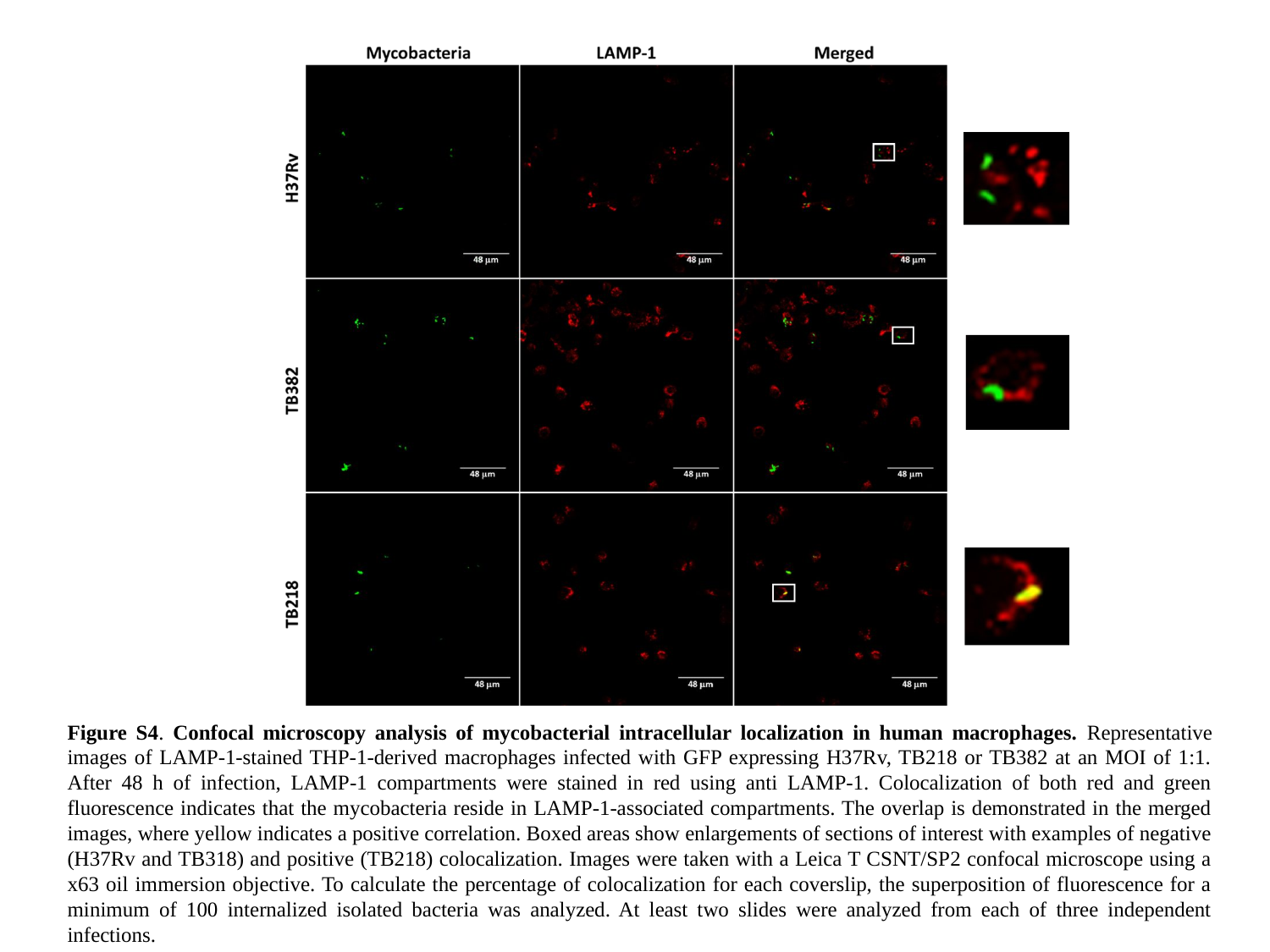

Figure S4. Confocal microscopy analysis of mycobacterial intracellular localization in human macrophages. Representative images of LAMP-1-stained THP-1-derived macrophages infected with GFP expressing H37Rv, TB218 or TB382 at an MOI of 1:1. After 48 h of infection, LAMP-1 compartments were stained in red using anti LAMP-1. Colocalization of both red and green fluorescence indicates that the mycobacteria reside in LAMP-1-associated compartments. The overlap is demonstrated in the merged images, where yellow indicates a positive correlation. Boxed areas show enlargements of sections of interest with examples of negative (H37Rv and TB318) and positive (TB218) colocalization. Images were taken with a Leica T CSNT/SP2 confocal microscope using a x63 oil immersion objective. To calculate the percentage of colocalization for each coverslip, the superposition of fluorescence for a minimum of 100 internalized isolated bacteria was analyzed. At least two slides were analyzed from each of three independent infections.
